# The practical clinical role of machine learning models with different algorithms in predicting prostate cancer local recurrence after radical prostatectomy

**DOI:** 10.1186/s40644-024-00667-x

**Published:** 2024-02-07

**Authors:** Chenhan Hu, Xiaomeng Qiao, Chunhong Hu, Changhao Cao, Ximing Wang, Jie Bao

**Affiliations:** Department of Radiology, the First Affiliated Hospital of Soochow University, 188#, Shizi Road, Suzhou, 215006 China

**Keywords:** Multiparametric Magnetic Resonance Imaging, Prostate Cancer, Local recurrence, Radiomics, Machine learning

## Abstract

**Background:**

The detection of local recurrence for prostate cancer (PCa) patients following radical prostatectomy (RP) is challenging and can influence the treatment plan. Our aim was to construct and verify machine learning models with three different algorithms based on post-operative mpMRI for predicting local recurrence of PCa after RP and explore their potential clinical value compared with the Prostate Imaging for Recurrence Reporting (PI-RR) score of expert-level radiologists.

**Methods:**

A total of 176 patients were retrospectively enrolled and randomly divided into training (*n* = 123) and testing (*n* = 53) sets. The PI-RR assessments were performed by two expert-level radiologists with access to the operative histopathological and pre-surgical clinical results. The radiomics models to predict local recurrence were built by utilizing three different algorithms (i.e., support vector machine [SVM], linear discriminant analysis [LDA], and logistic regression-least absolute shrinkage and selection operator [LR-LASSO]). The combined model integrating radiomics features and PI-RR score was developed using the most effective classifier. The classification performances of the proposed models were assessed by receiver operating characteristic (ROC) curve analysis.

**Results:**

There were no significant differences between the training and testing sets concerning age, prostate-specific antigen (PSA), Gleason score, T-stage, seminal vesicle invasion (SVI), perineural invasion (PNI), and positive surgical margins (PSM). The radiomics model based on LR-LASSO exhibited superior performance than other radiomics models, with an AUC of 0.858 in the testing set; the PI-RR yielded an AUC of 0.833, and there was no significant difference between the best radiomics model and the PI-RR score. The combined model achieved the best predictive performance with an AUC of 0.924, and a significant difference was observed between the combined model and PI-RR score.

**Conclusions:**

Our radiomics model is an effective tool to predict PCa local recurrence after RP. By integrating radiomics features with the PI-RR score, our combined model exhibited significantly better predictive performance of local recurrence than expert-level radiologists’ PI-RR assessment.

## Background

Radical prostatectomy (RP) is a common primary treatment choice for patients with low- and intermediate-risk prostate cancer (PCa) [1], while monitoring prostate-specific antigen (PSA) levels after surgery has been a standard approach for detecting any possible biochemical recurrence (BCR) [2]. Yet, the natural history of BCR after a surgical procedure is highly variable, and only a particular subset demonstrating specific clinicopathologic characteristics might be at higher risk of recurrence and benefit from salvage therapy [3]. Thus, BCR has become a well-established indication for choline or prostate-specific membrane antigen (PSMA) positron emission tomography/computed tomography (PET/CT) and multi-parametric magnetic resonance imaging (mpMRI), which is essential for detecting potential local recurrence or metastasis and deciding local salvage treatment [4–8]. However, studies have also shown that mpMRI can outperform choline or PSMA PET/CT in predicting local recurrence [9–11]. Thus, it is vital for patients experiencing BCR after RP to precisely assess post-operative mpMRI to detect any possible local recurrence lesion, which can significantly improve their clinical outcomes by tailoring the treatment plan.

A group of experts proposed the Prostate Imaging for Recurrence Reporting (PI-RR) assessment system to provide guidelines for standardizing image acquisition, interpretation, and scoring of mpMRI to detect local recurrence in PCa patients following RP or radiation therapy [12]. PI-RR scoring system aims to precisely locate and evaluate suspicious local recurrence lesions, which ultimately helps to personalize treatment plans [13, 14]. However, due to the subjectivity of radiologists and the ambiguity of some lesion criteria, the inter-observer agreement of the PI-RR assessment across different levels of readers remains questionable, requiring further evaluations for its predictive accuracy and clinical value [15]. In addition, the criteria employed to define different scores of each sequence have not yet gained universal acceptance, and the actual recurrence frequency of each PI-RR category remains uncertain, contributing to the potential requirement for clarification and adjustment of these criteria after prospective studies and randomized trials like Prostate Imaging Reporting and Data System (PI-RADS) [16]. It is also worth mentioning that the experience and accumulation of different radiologists significantly influence the score’s accuracy. The above-mentioned limitations have prompted a growing demand for innovative auxiliary techniques for analyzing post-RP mpMRI.

As a robust and relatively accurate image analysis technique, radiomics could create appropriate diagnosis and prognosis prediction models by extracting and analyzing high-dimensional features not seen by the naked eye [17]. Compared to qualitative imaging assessments conducted by radiologists, radiomics has some advantages, including stable calculation, moderate repeatability, and relative objectivity. Recent studies have shown radiomics analysis may be useful for PCa diagnosis, Gleason score classification, and biochemical recurrence prediction based on pre-operative MRI images [18–21]. Nevertheless, no study has explored the potential value of post-surgical mpMRI radiomics in detecting local recurrence of PCa patients, neglecting the importance of post-RP prostatic MRI for local recurrence evaluation. Thus, in this study, we developed and validated radiomics models with three algorithms based on post-operative mpMRI for local recurrence prediction in PCa patients who underwent RP. We further constructed a combined model by integrating radiomics features with the PI-RR score and compared the performance of machine learning models with the PI-RR assessment of expert-level radiologists to assess the potential value of these models in real-world clinical practice.

## Methods

### Patients

**Fig. 1:**
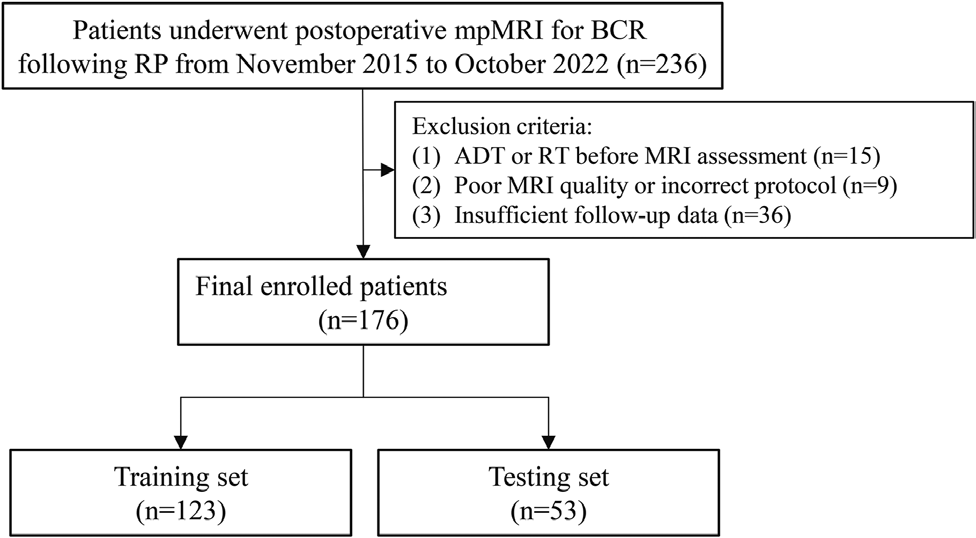
Study flow chart. mpMRI = multiparametric magnetic resonance imaging; BCR = biochemical recurrence; RP = Radical prostatectomy; ADT = androgen deprivation therapy; RT = radiotherapy

We comprehensively searched our institutional electronic database to identify PCa patients who underwent post-operative prostate mpMRI for clinically suspected local recurrence following RP between November 2015 and October 2022. Inclusion criteria were: (1) those who experienced BCR or PSA persistence following RP (two consecutive serum PSA values > 0.2 ng/mL following RP) [22]; (2) those who underwent standard prostate mpMRI for suspected local recurrence after RP. The exclusion criteria were the following: (1) androgen deprivation therapy (ADT) or radiotherapy (RT) before post-operative MRI assessment; (2) poor imaging quality or inappropriate MRI protocol; (3) insufficient follow-up data. The study flow chart is shown in Fig. 1.

Clinical data, including age, pre-operative PSA, follow-up PET-CT results, and PI-RADS score, were also collected from the electronic database of our institution. Histopathologic data were obtained from the surgical pathology reports, including the International Society of Urological Pathology Gleason scores (GS), pathologic T stage, perineural invasion (PNI), seminal vesicle invasion (SVI), and positive surgical margins (PSM).

The Institutional Ethics Review Board approved this retrospective study and waived the requirement for written informed consent due to the retrospective study design.

### MRI acquisition and analysis

The prostatic mpMRI examinations were performed using a 3.0T MRI scanner (Skyra; Siemens, Munich, Germany) with a pelvic phased-array surface coil without an endorectal coil. The prostate mpMRI protocol, including T1-weighted (T1WI), T2-weighted (T2WI) in three planes, diffusion-weighted imaging (DWI) and dynamic contrast-enhanced (DCE) T1WI, conformed to the PI-RR recommendations [13]. ADC images were calculated based on the DWI images of 50 and 1000 b-values using an extended single exponential fitting model. Next, the early enhancement phase (E2) of DCE images was selected for radiomics analysis following Nie K’s method [23], which specifically identifies this phase as occurring within 10 s of the appearance of contrast agents in the femoral arteries. The specific details of the examination protocol are displayed in Table 1.

**Table 1:** MRI sequences and parameters for radiomics analysis

Parameters	T2WI	DWI	DCE
Repetition time (ms)	6980.0	6540.0	4.2
Echo time (ms)	104.00	64.00	1.34
Layer thickness (mm)	3	3	3
Interlayer spacing (mm)	0	0	0
Field of view (mm × mm)	200 × 200	220 × 220	240 × 240
Imaging matrix	384 × 384	130 × 130	224 × 224
B-value (s/mm^2^)	/	50, 1000, 1500	/
FlipAngle	120	180	12
PixelSpacing (mm)	0.52 × 0.52	1.69 × 1.69	1.07 × 1.07

After T2WI, T1WI and DWI acquisitions, we administered a dose of gddiethylenetriaminepenta-acetic acid (0.05 mmol/kg; 3mL/sec; Magnevist, Bayer, Berlin, Germany) through a 20G antecubital intravenous line by using an MR-compatible injector (Spectris; Medrad, Pittsburgh, PA) to acquire DCE images. T2WI = T2-weighted imaging; T1WI = T1-weighted imaging; DWI = diffusion-weighted imaging; DCE = dynamic contrast-enhanced

All post-operative mpMRI were independently assessed by two expert-level radiologists (reader 1 with 10 years of professional experience, reader 2 with 15 years of professional experience in prostate MRI diagnosis) in compliance with PI-RR criteria [13]. All readers were aware of pre-operative clinical and surgical pathological data, including primary tumor location. Cases with indeterminate lesions or scores were assessed by a third experienced radiologist (reader 3 with more than 20 years of professional experience in prostate cancer imaging). In the present study, the lesion scored with the highest PI-RR in mpMRI was assessed if a case contained multiple lesions.

According to the PI-RR guidelines [13], the three-dimensional entire volume of interest (VOI) encompassing the whole suspicious lesion was manually contoured on axial slices of T2WI, DWI, ADC and early enhancement phase of DCE by reader 2, who participated in the PI-RR evaluation, using ITK-SNAP software (version 3.6.0). For individuals with a PI-RR score of 1, both DWI and DCE sequences showed no abnormal signal, and we delineated normal vesicourethral anastomosis. For patients with a PI-RR score of 2, the suspicious lesion was defined as the focus showing diffuse or heterogeneous enhancement in DCE images. For patients scoring 3–5, the lesion with the highest PI-RR score was delineated. The largest lesion was segmented if two or more lesions exhibited equally high PI-RR scores. Reader 3, with more than 20 years of professional experience, reviewed all annotations. The radiologists had access to the operative histopathological and pre-surgical clinical results while segmenting VOIs. To guarantee the intra-observer consistency of annotations, the segmentation procedure was repeated by reader 2 after an 8-week interval. Reader 1 also segmented all VOIs to evaluate inter-observer repeatability.

### Gold standard of reference

Based on previously reported reference standards [14], the criteria to define a post-operative mpMRI assessment as true-positive consisted of (1) a histologically confirmed positive result from biopsy specimens of the prostate or prostatectomy bed; (2) a volume enlargement detected by imaging modalities (including pelvic MRI, choline or gallium PSMA PET/CT) after more than 1 year of follow-up; (3) a volume shrinkage of a previously observed recurrent lesion at various imaging modalities or a reduction of PSA values following treatments (including ADT or salvage therapy) with a follow-up of > 2 years, restricted to patients with no signs of regional or distant metastasis on nuclear imaging (including bone scan, choline or gallium PSMA PET/CT).

The criteria for defining a post-operative mpMRI evaluation as true-negative consisted of [14]: (1) a biopsy-proven negative histopathological result obtained from prostatectomy bed or residual prostate; (2) a negative finding without tumor progression at various imaging modalities (including choline or gallium PSMA PET/CT or pelvic MRI) for more than 1 year of follow-up, accompanied by no rise of PSA levels for > 2 years.

### Radiomics feature extraction and selection

We respectively extracted 1781 radiomics features from each sequence, including T2WI, DWI, ADC and early enhancement phase of DCE, using the pyradiomics package in Python [24]. The extracted radiomics features contained shape, first-order and texture features from original and filtered images. We calculated texture features utilizing the gray-level co-occurrence matrix (GLCM), gray-level run length matrix (GLRLM), gray-level size zone matrix (GLSZM), gray-level dependence matrix (GLDM), and neighboring gray-tone difference matrix (NGTDM). The image transformation types included Wavelet, Laplacian of Gaussian (LOG), square, square root, logarithm, exponential, gradient, local binary pattern (2D), and local binary pattern (3D). The intra-observer and inter-observer consistency of lesion delineation were estimated with the intraclass correlation coefficient (ICC), and only radiomics features exhibiting both intra-observer and inter-observer ICC values > 0.80 were preserved for the following study.

We utilized FeAture Explorer (FAE) software (0.5.5) [25] to pre-process radiomics features and develop machine learning models. FAE is an open-source platform capable of extracting features, selecting features, constructing models, and visualizing results. First, the synthetic minority oversampling technique (SMOTE) was used to balance positive and negative samples of the training cohort. Second, we standardized the radiomics features by Z-score normalization, subtracting and dividing the mean value by the standard deviation for each feature. Third, the Pearson correction coefficient (PCC) analysis was utilized to reduce dimensionality. If the PCC of a feature pair surpassed 0.9, which means a high correlation between these two features, one of them was randomly eliminated. Finally, to filter significant radiomics features, we employed recursive feature elimination (RFE), which selects the best (or worst) features by iteratively constructing machine learning models for each feature. The feature selection procedure was carried out in the training set, with the number of selected features limited to a range of 1 to 20.

### Model development and validation

Employing the selected radiomics features, three prevalent machine learning models, based on support vector machine (SVM), linear discriminant analysis (LDA), and logistic regression-least absolute shrinkage and selection operator (LR-LASSO), were built to identify the classifier with the best prognostic prediction capability. Five-fold cross-validation was employed in the training cohort to determine the hyper-parameters of radiomics models. The hyper-parameters were adjusted in accordance with the model performance in the validation set. The area under the curve (AUC) obtained from the receiver operating characteristic (ROC) curve, sensitivity, specificity, accuracy, positive prediction value (PPV), and negative prediction value (NPV) of the three models were calculated to select the best radiomics model for the following analysis.

**Fig. 2:**
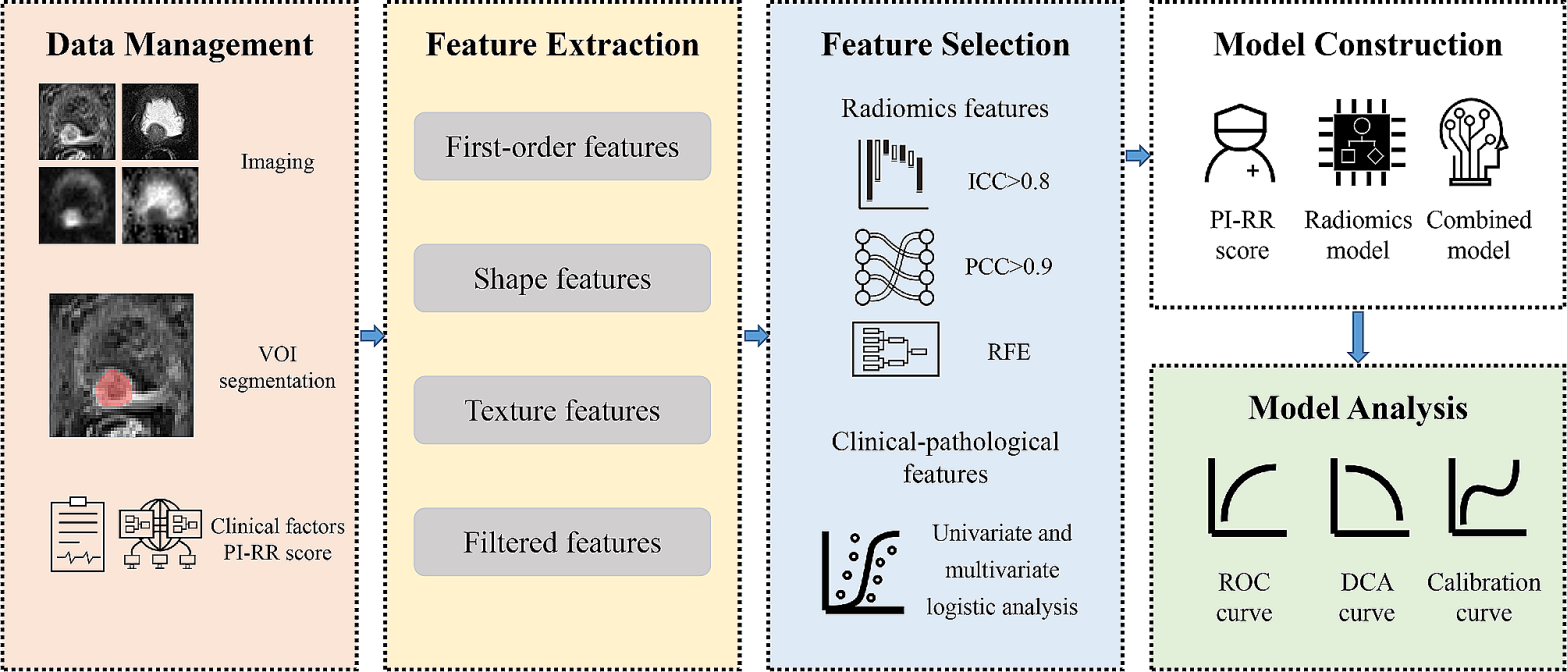
Imaging analysis and data flow of the research. VOI = volume of interest; PI-RR = Prostate Imaging for Recurrence Reporting system; ICC = intraclass correlation coefficient; PCC = Pearson correction coefficient; RFE = recursive feature elimination; ROC = receiver operating characteristic; DCA = decision curve analysis

First, we compared the predictive performance of the best radiomics model with the PI-RR assessment of expert-level radiologists to evaluate their ability to predict PCa local recurrence. Then, clinicopathologic features were entered into univariate and multivariable logistic regression analyses to estimate their predictive capability. The radiomics features obtained through RFE and clinicopathologic features selected through logistic regression analyses were evaluated for correlation. We removed features demonstrating high correlation (PCC > 0.9) to acquire the final features for combined model construction. To uniformly and objectively compare the predictive performance of all models, the machine learning algorithm that performed best in the radiomics models was chosen to construct the combined model by integrating significant clinicopathologic and radiomics features. Finally, we compared the combined model with the PI-RR score assessed by expert-level radiologists to explore if the combined model could further improve the predictive level. Figure 2 displays the entire workflow of this study.

### Statistical analysis

SPSS 26.0 software, Python software (version 3.5.6) and R software (version 3.6.3) were used for statistical analysis. Continuous variables were represented as the mean ± standard deviation or median with interquartile range (IQR) in accordance with the normality test, while categorical variables were reported as frequency and proportions. We employed the Shapiro–Wilk test to verify the normality of features. The independent-sample t-test or Mann–Whitney U test was applied to compare quantitative parameters, and the chi-square test was utilized to compare qualitative parameters.

ROC curves and corresponding AUC values assessed all models. In accordance with previous findings [14, 26], PI-RR ≥ 3 was used to define a positive post-operative mpMRI assessment. The best cutoff values of machine learning models were determined according to the maximization of the Youden Index in the training cohort. The sensitivity, specificity, accuracy, PPV, and NPV of all models were calculated for predictive performance comparison. The DeLong test was employed to compare the AUCs of all models. Decision curve analysis (DCA), which estimated the net benefits at varying threshold probabilities, was used to evaluate the clinical applicability of the PI-RR system, radiomics, and combined models. The calibration curve was plotted to assess the calibration ability of the combined model. A two-tailed *p*-value < 0.05 represented statistical significance.

## Results

### Clinical characteristics

A total of 176 eligible patients were included and were randomly allocated to the training (*n* = 123) and testing (*n* = 53) sets using a 7:3 ratio. There was no significant difference between the training and testing sets concerning age, PSA, Gleason score, T stage, SVI, PNI and PSM (all *p* > 0.05). A comprehensive overview of clinical characteristics for the entire study cohort is provided in Table 2.

**Table 2:** Patient characteristics

Characteristics	Training set *n* = 123	Testing set *n* = 53	P value
Age (years, mean ± SD)	69.4 ± 6.7	70.1 ± 6.6	0.938
Pre-operative PSA level [ng/mL, median (IQR)]	19.5 (6.5–32.5)	24.8 (7.4–42.2)	0.835
The time between RP and MRI [months, median (IQR)]	10.3 (2.5–18.1)	12.3 (3.6–21.1)	0.896
Follow-up time [months, median (IQR)]	59.0 (35.5–82.5)	46.0 (30.0–61.0)	0.343
Surgical Gleason score [n, (%)]			0.816
6	6 (4.9%)	3 (5.7%)	
7 (3 + 4)	29 (23.6%)	9 (17.0%)	
7 (4 + 3)	35 (28.5%)	18 (34.0%)	
8	19 (15.4%)	10 (18.9%)	
9–10	34 (27.6%)	13 (24.5%)	
Pathologic T stage [n, (%)]			0.168
T2a/b	8 (6.5%)	9 (17.0%)	
T2c	42 (34.1%)	16 (30.2%)	
T3a	35 (28.5%)	12 (22.6%)	
T3b	19 (15.4%)	11 (20.8%)	
T4	19 (15.4%)	5 (9.4%)	
SVI [n, (%)]	29 (23.6%)	13 (24.5%)	0.892
PNI [n, (%)]	60 (48.8%)	19 (35.8%)	0.114
PSM [n, (%)]	59 (48.0%)	29 (54.7%)	0.411
Local recurrence evident [n, (%)]	38 (30.9%)	16 (30.2%)	0.926
Pre-operative PI-RADS score [n, (%)]			0.622
1–2	7 (5.7%)	1 (1.9%)	
3	13 (10.6%)	5 (9.4%)	
4	34 (27.6%)	13 (24.5%)	
5	69 (56.1%)	34 (64.2%)	
Post-operative PI-RR score [n, (%)]			0.473
1	76 (61.8%)	31 (58.5%)	
2	10 (8.1%)	8 (15.1%)	
3	10 (8.1%)	5 (9.4%)	
4	15 (12.2%)	3 (5.7%)	
5	12 (9.8%)	6 (11.3%)	

SD = standard deviation; IQR = interquartile range; PSA = prostate specific antigen; RP = radical prostatectomy; SVI = seminal vesicle invasion; PNI = perineural invasion; PSM = positive surgical margins; PI-RADS = Prostate Imaging Reporting and Data System; PI-RR = Prostate Imaging for Recurrence Reporting

### PI-RR Assessment of mpMRI after RP

**Fig. 3:**
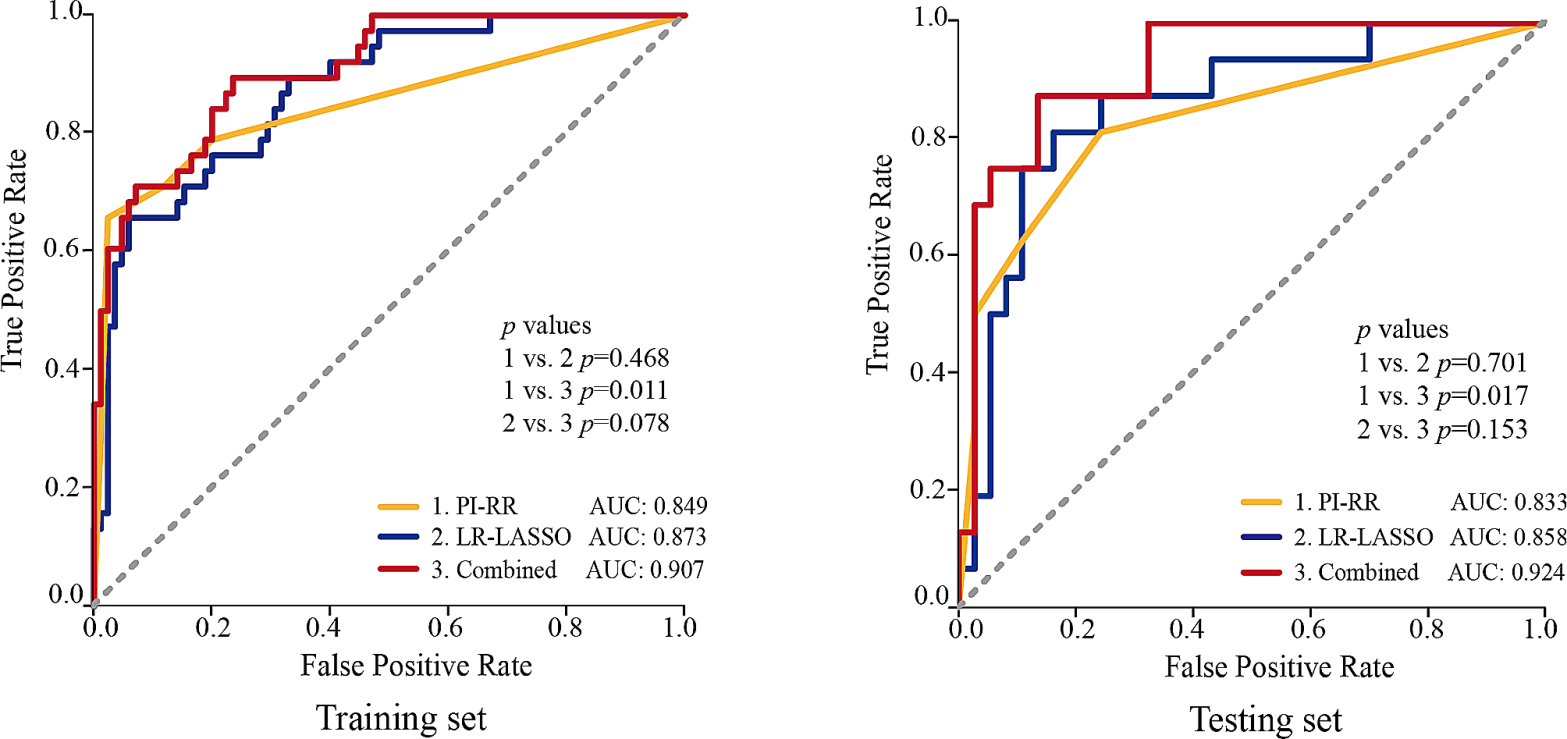
The comparison of PI-RR score, LR-LASSO model, and combined model in predicting PCa local recurrence in the training and testing sets. LR-LASSO = logistic regression-least absolute shrinkage and selection operator; PI-RR = Prostate Imaging for Recurrence Reporting system; AUC = area under the curve

The univariate logistic regression analysis revealed that the PI-RR score, PI-RADS category and surgical Gleason score were significantly associated with PCa local recurrence. Yet, the multivariate logistic regression analysis showed that only the PI-RR score (odds ratio [OR] = 3.283; 95% confidence interval [CI]: 2.175–4.956; *p* < 0.001) was the independent risk factor for predicting local recurrence (Table 3). The performance of the PI-RR score in predicting local recurrence following RP is presented in Fig. 3. In the testing set, PI-RR yielded an AUC of 0.833 (95%CI: 0.708–0.958); the sensitivity and specificity in predicting local recurrence were 0.625 (10/16) and 0.892 (33/37), respectively.

**Table 3:** Univariate and multivariate logistic regression analyses of clinical features

Variable	Univariate analysis		Multivariate analysis	
	Odds ratio (95% CI)	P-value	Odds ratio (95% CI)	P-value
Age	1.040 (0.980–1.103)	0.199		
Pre-operative PSA	1.004 (0.996–1.011)	0.342		
Surgical Gleason score	1.583 (1.137–2.203)	0.006	1.503 (0.923–2.448)	0.102
Pathological T stage	1.066 (0.770–1.475)	0.701		
SVI	1.846 (0.776–4.390)	0.165		
PNI	0.921 (0.428–1.982)	0.834		
PSM	1.311 (0.609–2.821)	0.489		
PI-RADS	2.542 (1.354–4.771)	0.004	2.116 (0.901–4.970)	0.085
PI-RR	3.283 (2.232–4.831)	< 0.001	3.283 (2.175–4.956)	< 0.001

95% CI = 95% confidence interval; PSA = prostate specific antigen; SVI = seminal vesicle invasion; PNI = perineural invasion; PSM = positive surgical margins; PI-RADS = Prostate Imaging Reporting and Data System; PI-RR = Prostate Imaging for Recurrence Reporting

### Construction and validation of radiomics models

**Fig. 4:**
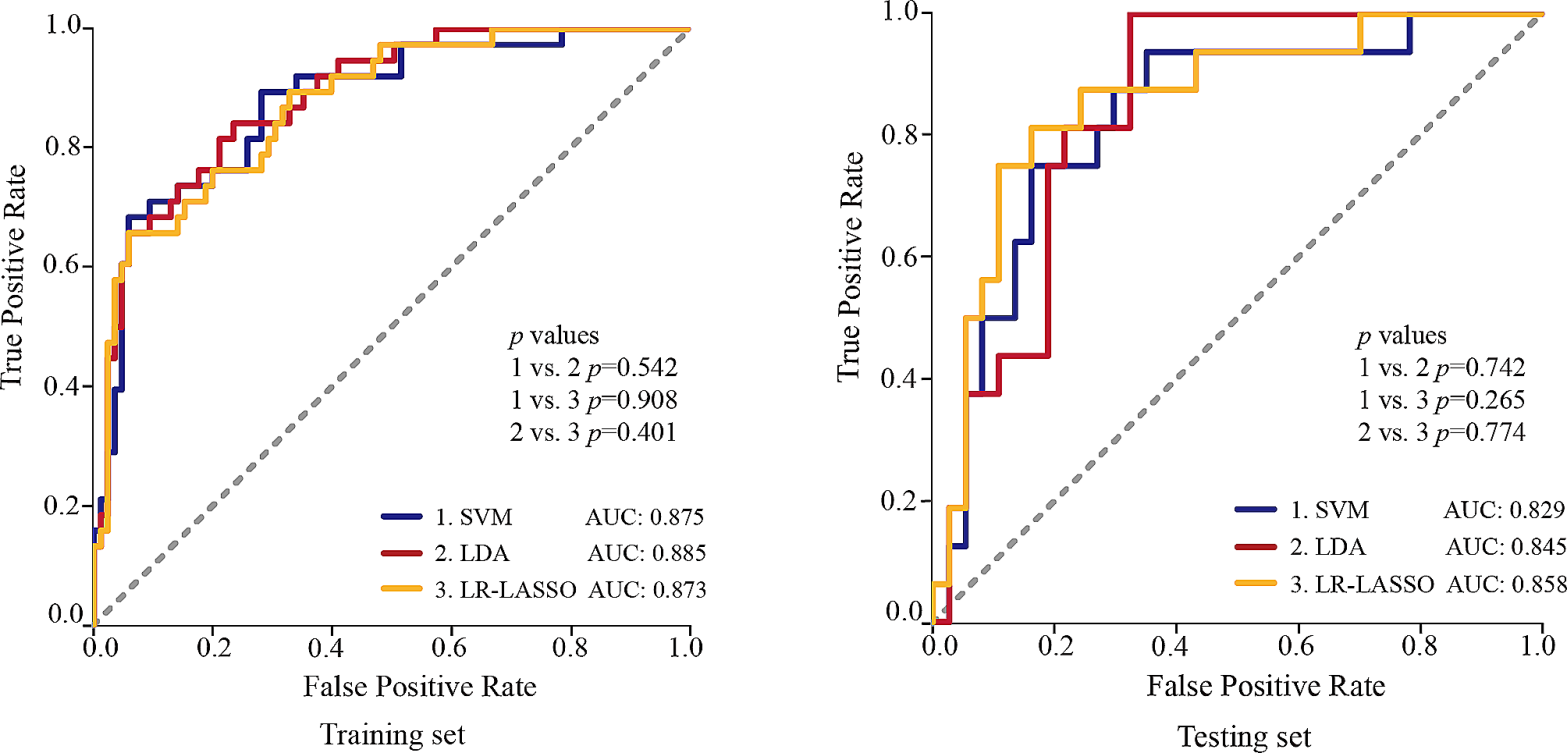
The comparison of the predictive performances of radiomics models using three different machine learning algorithms in predicting PCa local recurrence in the training and testing sets. SVM = support vector machine; LDA = linear discriminant analysis; LR-LASSO = logistic regression-least absolute shrinkage and selection operator; AUC = area under the curve

The mean intra-observer and inter-observer ICCs were separately 0.896 and 0.841, suggesting good reliability of lesion delineation and feature extraction. Following feature reduction and selection, 14 radiomics features were preserved and employed for subsequent model construction (Table 4). The performances of three different radiomics models, based on LR-LASSO, SVM, and LDA algorithms, are displayed in Fig. 4 and Table 5. The performance of the radiomics model based on LR-LASSO was the best among all radiomics models in the testing set. LR-LASSO model yielded an AUC of 0.858 (95% CI: 0.746–0.971) with a sensitivity of 0.750 (12/16) and specificity of 0.892 (33/37) in the testing set. The AUC of the LR-LASSO model was numerically higher than SVM and LDA models (all *p* > 0.05). Consequently, we chose the radiomics model based on the LR-LASSO algorithm, which yielded the highest AUC for the following application.

**Table 4:** Selected features of radiomics model and combined model

Radiomic model	Combined model
T2WI_log-sigma-1-mm-3D_ngtdm_Contrast	T2WI_log-sigma-1-mm-3D_ngtdm_Contrast
ADC_exponential_glrlm_LongRunLowGrayLevelEmphasis	ADC_exponential_glrlm_LongRunLowGrayLevelEmphasis
ADC_exponential_glszm_SmallAreaEmphasis	ADC_exponential_glszm_SmallAreaEmphasis
DWI_exponential_glszm_SmallAreaLowGrayLevelEmphasis	DWI_exponential_glszm_Zone%
DWI_exponential_glszm_Zone%	DWI_gradient_glrlm_LongRunHighGrayLevelEmphasis
DWI_gradient_glrlm_LongRunHighGrayLevelEmphasis	DWI_gradient_glrlm_LongRunLowGrayLevelEmphasis
DWI_gradient_glrlm_LongRunLowGrayLevelEmphasis	DWI_square_glrlm_RunVariance
DWI_square_glrlm_RunVariance	DCE_lbp-3D-m1_firstorder_RootMeanSquared
DWI_wavelet-HHL_firstorder_Mean	DCE_lbp-3D-m2_firstorder_Median
DCE_lbp-3D-m1_firstorder_RootMeanSquared	DCE_wavelet-LHL_firstorder_Median
DCE_lbp-3D-m2_firstorder_Median	PI-RR
DCE_lbp-3D-m2_firstorder_Skewness	
DCE_wavelet-LHL_firstorder_Mean	
DCE_wavelet-LHL_firstorder_Median	

T2WI = T2-weighted imaging; DWI = diffusion-weighted imaging; ADC = apparent diffusion coefficient; DCE = dynamic contrast-enhanced; PI-RR = Prostate Imaging for Recurrence Reporting

**Table 5:** Predictive performance of different models in training and testing sets

Models		AUC (95% CI)	Accuracy	Sensitivity	Specificity	PPV	NPV
LR-LASSO	Training	0.873 (0.807–0.938)	0.789	0.763	0.800	0.630	0.883
	Testing	0.858 (0.746–0.971)	0.849	0.750	0.892	0.750	0.892
SVM	Training	0.875 (0.808–0.942)	0.813	0.737	0.847	0.683	0.878
	Testing	0.829 (0.708–0.951)	0.755	0.500	0.865	0.615	0.800
LDA	Training	0.885 (0.824–0.946)	0.821	0.737	0.859	0.700	0.880
	Testing	0.845 (0.742–0.948)	0.717	0.500	0.811	0.533	0.790
PI-RR	Training	0.849 (0.770–0.929)	0.829	0.710	0.882	0.730	0.872
	Testing	0.833 (0.708–0.958)	0.811	0.625	0.892	0.714	0.846
Combined	Training	0.907 (0.853–0.961)	0.805	0.895	0.765	0.630	0.942
	Testing	0.924 (0.851–0.997)	0.868	0.875	0.865	0.737	0.941

AUC = area under the curve; CI = confidence interval; PPV = positive predictive value; NPV = negative predictive value;

LR-LASSO = logistic regression-least absolute shrinkage and selection operator; SVM = support vector machine; LDA = linear discriminant analysis; PI-RR = Prostate Imaging for Recurrence Reporting

Notably, the AUC of the radiomics model based on LR-LASSO was numerically higher than the PI-RR score, but there was no statistically significant difference between the LR-LASSO model and PI-RR score in the testing set (*p* = 0.701). This suggested that the performance of the radiomics model is comparable to that of the PI-RR score assessed by expert-level radiologists and may be useful for predicting PCa local recurrence.

### Development and verification of combined model

We further developed a combined model by integrating radiomics features and PI-RR score to evaluate its potential value in predicting the local recurrence of PCa (Table 4). The predictive performances of the PI-RR score, radiomic model based on LR-LASSO, and combined model are displayed in Fig. 3; Table 5. The combined model achieved the highest AUC in predicting local recurrence in the testing cohort (AUC = 0.924, 95%CI: 0.851–0.997). In the testing set, the sensitivity and specificity in predicting local recurrence were 0.875 (14/16) and 0.865 (32/37), respectively. Notably, the AUC of the combined model was significantly higher than the PI-RR score (AUC: 0.924 vs. 0.833) (*p* = 0.017) in the testing set, but no significant difference was observed between the combined model and radiomics model (AUC: 0.924 vs. 0.858) (*p* = 0.153). In addition, the combined model exhibited a substantial increase in sensitivity at a slight cost of specificity in comparison with the PI-RR score (sensitivity: 0.875 vs. 0.625; specificity: 0.865 vs. 0.892).

**Fig. 5:**
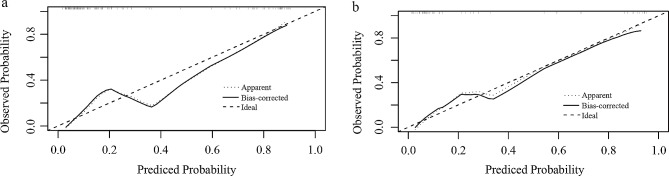
The calibration curve of combined model in the training (a) and testing (b) sets

**Fig. 6:**
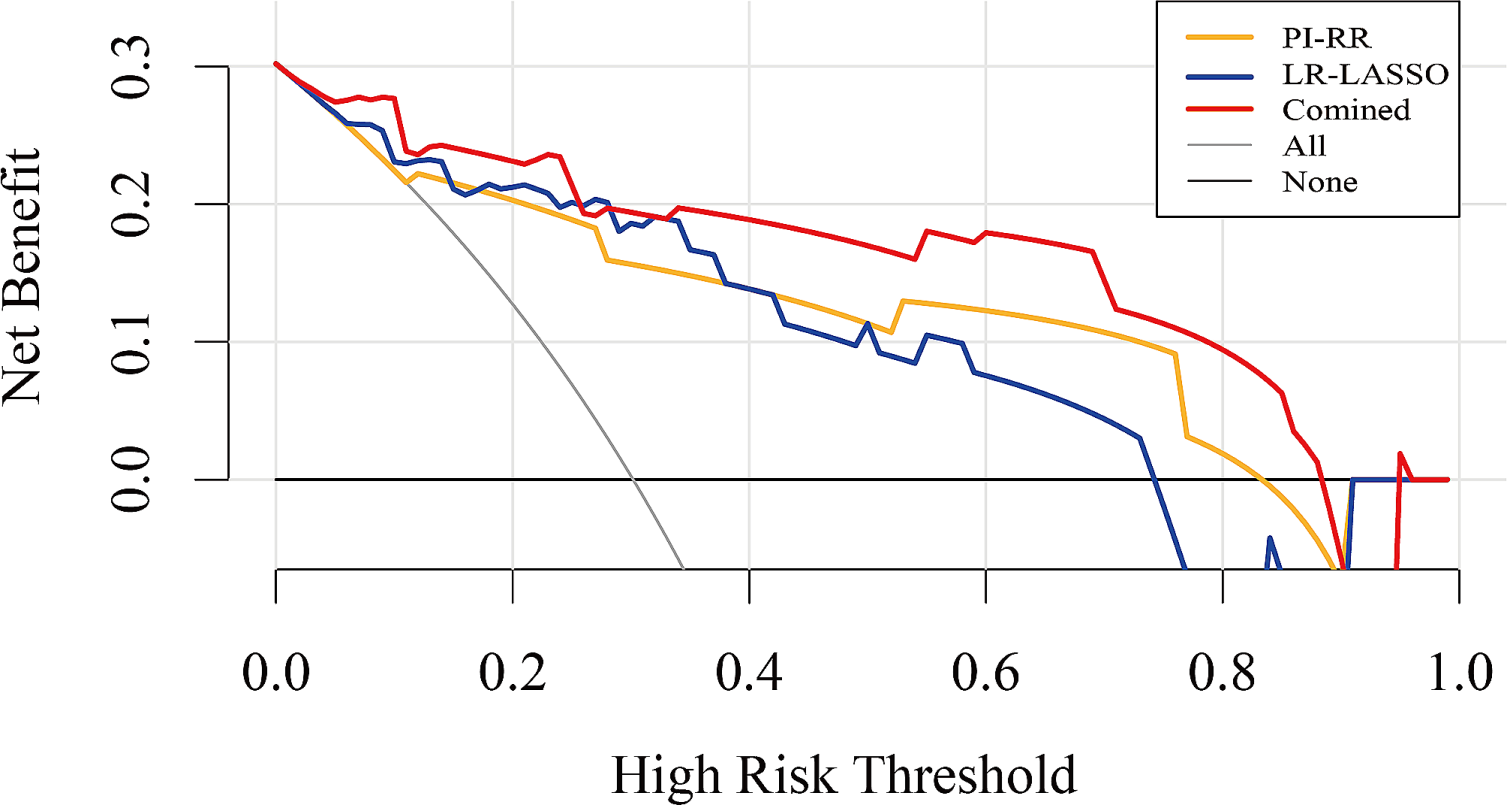
Decision curve analysis (DCA) for the PI-RR score, radiomics model and combined models in the testing set. PI-RR = Prostate Imaging for Recurrence Reporting system; LR-LASSO = logistic regression-least absolute shrinkage and selection operator

The calibration curve revealed that the combined model demonstrated good concordance between prediction and observation (Fig. 5), and the Hosmer–Lemeshow test indicated good calibration for the combined model in both training (*p* = 0.330) and testing (*p* = 0.671) sets. The DCAs of the PI-RR score, radiomic model and combined model in the testing set are displayed in Fig. 6. While all models achieved higher net benefits than the treat-all or treat-none protocol across most range of threshold probabilities, the combined model demonstrated the most substantial net benefit, highlighting the clinical utility of the model.

## Discussion

Assessment of post-operative mpMRI can accurately detect PCa local recurrence after RP, which is of essential importance for estimating long-term prognosis and directing post-operative administration of PCa patients [14, 27]. PI-RR score obtained by mpMRI is a promising tool for the standardization of the assessment of patients who underwent RP (PI-RR scores of 1 and 2 are assigned to lesions with a very low and low likelihood of recurrence, respectively; PI-RR 3 is assigned if the presence of recurrence is uncertain; PI-RR 4 and 5 are assigned for a high and very high likelihood of recurrence) [13]. Recently, PI-RR preliminarily displayed its ability to improve the detection and characterization of suspicious PCa local recurrence lesions. Pecoraro et al. [14] reported that the PI-RR assessment system provides structured, reliable, and precise evaluation of suspicious local recurrence foci, with an AUC of 0.80–0.88, sensitivity of 0.59–0.83, and specificity of 0.87–1.00. Ciccarese et al. [26] found that the predictive ability of the PI-RR system was generally better than PET/CT scans for PCa local recurrence. The accuracy reached 0.68 but was influenced by the PSA values. Park et al. [28] proved that the PI-RR DCE score is associated with adverse clinic-pathologic characteristics and could predict 1-year BCR after RP. In this study, we retrospectively enrolled 176 patients and randomly divided them into training and testing sets, and two expert-level radiologists performed the PI-RR assessments. Our research findings about PI-RR were consistent with previous studies. The PI-RR yielded an AUC of 0.833 (95%CI: 0.708–0.958) in the testing set. In the testing set, its sensitivity and specificity in predicting local recurrence were 0.625 (10/16) and 0.892 (33/37), respectively. These findings suggested that the PI-RR system may optimize post-operative management by improving evaluation precision and customizing treatments. However, the PI-RR assessment system has the following limitations: first, the inter-reader reproducibility and practical value of the PI-RR system are still uncertain, lacking validations from prospective and multi-center research. Second, as demonstrated by our data and Pecoraro’s study [14], PI-RR had excellent specificity but only moderate sensitivity in predicting local recurrence, suggesting that a negative assessment could not completely exclude local recurrence and these patients still require monitoring. Third, the criteria employed to define different scores for each sequence have not yet been universally accepted, contributing to the potential requirement for clarification and adjustment of these criteria after prospective studies and randomized trials like Prostate Imaging Reporting and Data System (PI-RADS) [16]. Fourth, the accuracy of the scoring system is significantly influenced by the experience and expertise of the individual radiologist involved. Lastly, the PI-RR evaluation algorithm was established through professional consensus, and the real recurrence frequency of each PI-RR category remained uncertain [13]. Hence, despite the great progress made by the PI-RR system, thse limitations highlight the need for novel methodologies to be developed for assessing post-operative mpMRI.

In this study, we first constructed and validated machine learning models using three different classifiers for local recurrence evaluation and compared them with the PI-RR score obtained by expert-level radiologists. Unlike previous studies [14, 26, 28], which focused on the predictive efficacy of radiologists, radiomics, a semi-automatic quantitative image analysis method, was employed to predict PCa local recurrence. In our study, the features of the radiomics model and combined model were mainly composed of first-order features from DCE images and texture features from DWI and ADC images, indicating the intensity statistics from DCE images and the lesion heterogeneity information from DWI and ADC images have a key role in assessing suspicious local recurrence lesion. This finding is consistent with the PI-RR proposal, of which the final score is mainly generated with DWI and DCE images [13]. Besides, the radiomics and combined models did not utilize any feature extracted from original images, suggesting radiomics features from transformed images were more stable than those from original images in the evaluation of post-operative mpMRI.

Radiomics has been proven as an effective and valuable tool for diagnosis, risk stratification, and prognosis prediction in the field of prostatic MRI. Zheng et al. [29] argued that bpMRI-based radiomics is an accurate and stable tool to predict pelvic lymph node invasion of PCa patients. Shiradkar et al. [30] successfully employed radiomics features and clinical characteristics to predict BCR after RP. While most of the prior studies explored radiomics for assessing pre-operative MRI images, this is the first study that focused on the radiomics analyses of post-operative mpMRI sequences and further compared the predictive performance of radiomics models and radiologists for PCa local recurrence [31]. Our research evaluated local recurrence using three machine-learning algorithms, including LR-LASSO, SVM and LDA classifiers. The LR-LASSO algorithm showed the best predictive performance and demonstrated similar predictive ability with the PI-RR assessment of experts. To the best of our knowledge, this is the first study that explored the value of different radiomics models for predicting PCa local recurrence based on post-operative mpMRI.

By integrating the PI-RR score with radiomics features, the combined model exhibited a significantly higher AUC value than expert-level radiologists’ PI-RR assessment. In addition, compared to the PI-RR score, the combined model showed substantially higher sensitivity at a slight cost of specificity. Thus, it could be inferred that by combining qualitative manual evaluations and quantitative radiomics analyses together, we could achieve a more precise prediction of PCa local recurrence. The combined model may be a promising tool for predicting PCa local recurrence after RP and assisting clinical decision-making.

In this study, an assessment of various clinicopathological variables was conducted to identify potential predictors of local recurrence. Although the univariate logistic regression analysis showed that surgical Gleason score, PI-RADS, and PI-RR score were associated with local recurrence, the PI-RR score was the only risk factor remaining significant in the multivariate logistic regression analysis. Other clinical variables, such as pre-operative PSA, SVI, and PSM, were excluded from the model construction, which is consistent with the work of Pecoraro et al. [14]. This explains why an innovative clinical model was missing in our study, and the poor predictive performances of traditional clinical variables made it necessary to invent new tools for local recurrence prediction.

The present study has several limitations. First, as a single-center and retrospective study, future multi-center and prospective studies are needed to validate the generalizability and accuracy of our model. Second, we only utilized the early enhancement phase of DCE in our work, neglecting other phases of DCE images. Further studies are needed to investigate whether the radiomics features of other DCE phases could improve model performance. Third, the accuracy and subjectivity problems associated with manual VOI delineation highlight the need for automated segmentation based on deep learning techniques.

## Conclusions

The performance of the radiomics model based on LR-LASSO was comparable to PI-RR scoring of expert-level radiologists in predicting PCa local recurrence after RP. Most notably, by integrating radiomics features with PI-RR score, our combined model exhibited better performance in predicting local recurrence compared to PI-RR scored by expert-level radiologists. Hence, this new combined model can potentially improve the predictive performance of PI-RR assessed by expert-level radiologists and help clinicians tailor treatments for post-operative patients.

## Data Availability

The datasets used and/or analyzed during the current study are available from the corresponding author on reasonable request.

## Abbreviations

ADC: Apparent diffusion coefficient
AUC: Area under the curve
BCR: Biochemical recurrence
DWI: Diffusion-weighted imaging
DCA: Decision curve analysis
GS: Gleason scores
ICC: Intraclass correlation coefficient
LDA: Linear discriminant analysis
LR-LASSO: Logistic regression-least absolute shrinkage and selection operator
PCC: Pearson correction coefficient
PCA: Prostate cancer
PET/CT: Positron emission tomography/computed tomography
PNI: Perineural invasion
PSM: Positive surgical margins
PSA: Prostate specific antigen
PV: Prostate volume
PI-RADS: Prostate Imaging Reporting and Data System
PI-RR: Prostate Imaging for Recurrence Reporting
PVT: Prostate volume threshold
RFE: Recursive feature elimination
ROC: Receiver operating characteristic
RP: Radical prostatectomy
SVI: Seminal vesicle invasion
SVM: Support vector machine
